# Repeated Measurements of Cardiac Troponin T and N-Terminal Pro-B-Type Natriuretic Peptide to Assess Long-Term Mortality Risk in Subjects with Osteoarthritis

**DOI:** 10.3390/biom11020230

**Published:** 2021-02-05

**Authors:** Martin Rehm, Gisela Büchele, Rolf Erwin Brenner, Klaus-Peter Günther, Hermann Brenner, Wolfgang Koenig, Dietrich Rothenbacher

**Affiliations:** 1 Institute of Epidemiology and Medical Biometry, Ulm University, 89081 Ulm, Germany; martin.rehm@uni-ulm.de (M.R.); gisela.buechele@uni-ulm.de (G.B.); koenig@dhm.mhn.de (W.K.); 2 Division for Biochemistry of Joint and Connective Tissue Diseases, Department of Orthopedics, Ulm University, 89081 Ulm, Germany; rolf.brenner@uni-ulm.de; 3 University Center of Orthopedic and Trauma Surgery, Technical University of Dresden, 01307 Dresden, Germany; klaus-peter.guenther@uniklinikum-dresden.de; 4 Division of Clinical Epidemiology and Aging Research, German Cancer Research Center (DKFZ), 69120 Heidelberg, Germany; h.brenner@dkfz-heidelberg.de; 5 Division of Preventive Oncology, German Cancer Research Center (DKFZ) and National Center for Tumor Diseases (NCT), 69120 Heidelberg, Germany; 6 German Cancer Consortium (DKTK), German Cancer Research Center (DKFZ), 69120 Heidelberg, Germany; 7 Deutsches Herzzentrum München, Technische Universität München, 80636 Munich, Germany; 8 German Centre for Cardiovascular Research (DZHK), Partner Site Munich Heart Alliance, 80802 Munich, Germany

**Keywords:** high-sensitivity cardiac troponin T (hs-cTnT), N-terminal pro-B-type natriuretic peptide (NT-proBNP), repeated measurements, cohort study, osteoarthritis, mortality

## Abstract

Osteoarthritis (OA) is associated with higher cardiovascular mortality risk. High-sensitivity cardiac troponin T (hs-cTnT) and N-terminal pro-B-type natriuretic peptide (NT-proBNP) are well-characterized prognostic cardiac markers. We aimed to describe the changes in biomarkers measured one year apart in a cohort of 347 subjects with OA who underwent hip or knee replacement surgery in 1995/1996 and to analyze the prognostic value of repeated measurements for long-term mortality. During a median follow-up of 19 years, 209 (60.2%) subjects died. Substantial changes in cardiac biomarkers, especially for NT-proBNP, and an independent prognostic value of NT-proBNP for long-term mortality were found for both baseline measurement concentration (hazard ratio (HR) 1.32, 95% confidence interval (CI) (1.13–1.55)) and follow-up measurement concentration (HR 1.39, 95% CI 1.18–1.64) (all HR per standard deviation increase after natural log-transformation). Baseline concentrations were correlated with follow-up concentrations of NT-proBNP and no longer showed prognostic value when included simultaneously in a single model (HR 1.08, 95% CI 0.86–1.37), whereas the estimate for the one-year measurement remained robust (HR 1.31, 95% CI 1.04–1.66). Therefore, no significant additional benefit of repeated NT-proBNP measurements was found in this cohort, facilitating the use of a single NT-proBNP measurement as a stable prognostic marker.

## 1. Introduction

Cardiac biomarkers such as the N-terminal pro-B-type natriuretic peptide (NT-proBNP) and high-sensitivity troponins are valuable and well-characterized diagnostic and prognostic markers in cardiovascular medicine. Their prognostic value has been demonstrated both in the general population [1,2,3,4,5,6] and in high-risk cardiovascular cohorts [7,8,9,10]. In addition to measuring these markers at one point in time, repeated measurements and the evaluation of changes during certain periods may improve the risk prediction for adverse outcomes such as cardiovascular events or total mortality.

Osteoarthritis (OA) is a common musculoskeletal disorder. Its prevalence increases with age and it is associated with functional disabilities and joint pain [11]. Usually, arthroplasty is an established therapy to restore functional capacity and relieve pain. Patients with OA have a higher prevalence of cardiovascular diseases [12,13] (especially coronary artery disease and heart failure) and subsequently also an increased risk of death from cardiovascular causes compared to the general population [14,15]. Premature death from cardiovascular disease can potentially be prevented by suitable timely interventions (balanced diet/physical activity, medication, invasive measures). For this purpose, it is very important to identify at-risk individuals early on and to take appropriate measures at an early stage. However, the temporal changes of established cardiac markers within one year after arthroplasty and their prognostic value for adverse endpoints have rarely been investigated.

This study aimed to describe the one-year changes in NT-proBNP and high-sensitivity cardiac troponin T (hs-cTnT) in subjects with OA of the hip or knee and to analyze the prognostic value of the repeated measurements and changes on long-term mortality.

## 2. Materials and Methods

The Ulm Osteoarthritis Study is a multi-center prospective observational cohort study involving subjects who underwent unilateral total hip or knee replacement due to advanced OA between January 1995 and December 1996. All subjects were Caucasian and under 76 years of age at baseline. Subjects with malignant tumors, inflammatory diseases, corticosteroid medication, or previous hip or knee replacement were excluded. A total of 809 eligible subjects were included in the study cohort and underwent baseline examinations in Ulm, Augsburg, or Stuttgart (three cities in Southern Germany). A detailed description of the study methods was published previously [16,17]. The study was approved by the local ethics committee of the University of Ulm (No. 40/94 and 164/14) and the written consent of the subjects was given at the time of enrolment in the study.

**Figure 1:**
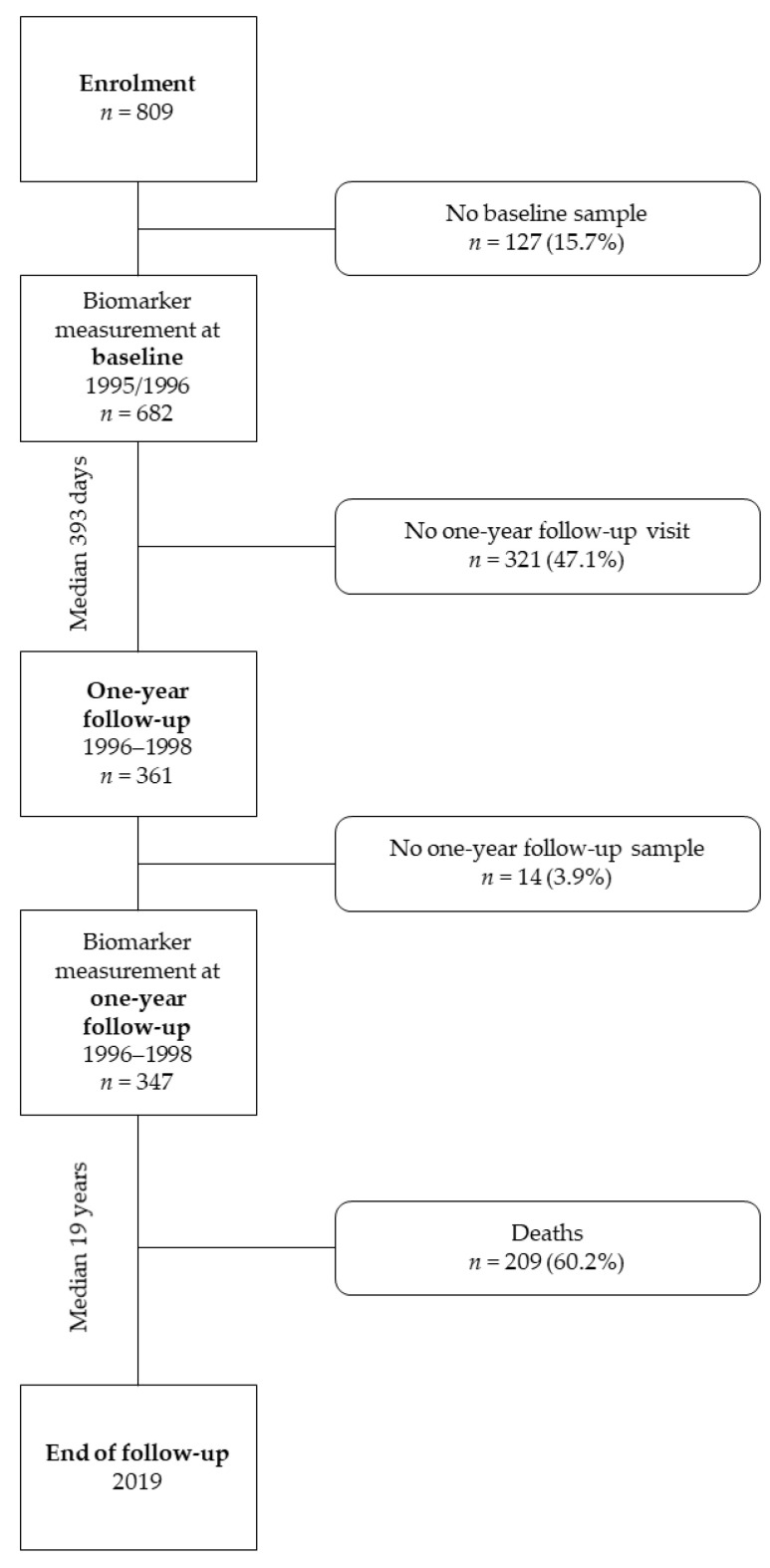
Study flow chart.

The current analysis included subjects who had hs-cTnT and NT-proBNP measurements at the baseline visit before arthroplasty and the scheduled one-year follow-up visit. This left *n* = 347 (42.9%) subjects for this evaluation (Figure 1).

In addition to demographic data (age, sex, smoking status, weight, and height), detailed information on the history of self-reported physician-diagnosed comorbidities (e.g., diabetes mellitus, hypertension, myocardial infarction, and heart failure) was collected in standardized interviews. Weight and height of the subjects were determined during the interview and used to calculate the body mass index (BMI).

Total cholesterol, triglycerides, creatinine, and uric acid levels were determined in non-fasting serum samples collected preoperatively by standard venipuncture and analyzed in a central laboratory at the time of baseline recruitment using routine methods. High-sensitivity C-reactive protein (hs-CRP) was measured with a commercial kit (NA-Latex-CRP, Behring Werke, Marburg, Germany), calibrated with the WHO reference standard 85/506. The glomerular filtration rate (eGFR) was estimated using the creatinine-based formula of the Chronic Kidney Disease Epidemiology Collaboration (CKD-EPI) [18].

NT-proBNP and hs-cTnT were measured in 2019 by a commercial electrochemiluminescence immunoassay (ECLIA; Cobas Elecsys 411, Roche, Mannheim, Germany) in serum samples collected at baseline and one year later and stored at −80 °C until analysis. The inter-assay coefficient of variation (CV) for NT-proBNP was < 5% during the test period and the limit of detection (LoD) was 5.0 ng/L. For hs-cTnT measurements, the detection limit was also 5.0 ng/L with CVs of 3.6% and 2.9% at concentrations of 42.0 and 2.8 ng/L, respectively. All values below the detection limit were reported as < 5.0 ng/L. During the same period, serum cystatin C was measured by immunonephelometry on a Behring Nephelometer II (CV 2.9–3.2%).

The primary endpoint of this evaluation was all-cause mortality. Registration authorities were contacted to obtain survival status and exact date of death if the subject was deceased. For 99.4% of subjects, survival status information could be obtained with the last update on June 11, 2019. The follow-up time was calculated in days, starting with the date of the one-year follow-up visit (where the second biomarker measurement was performed) and ending with the date of death. Non-deceased subjects were censored at the end of the maximum 23-year follow-up period.

Discrete variables are presented as numbers and percentages, continuous variables as medians and interquartile range (IQR). Due to the skewed distributions of NT-proBNP and hs-cTnT concentrations, the median concentrations were calculated for the different covariate levels. Undetectable NT-proBNP and hs-cTnT measurements (< 5.0 ng/L) were set to 2.5 ng/L (half the LoD of the assay). For further analysis, the NT-proBNP and hs-cTnT concentrations were natural log-transformed (ln).

In addition, crude bivariate associations between the clinical variables listed in Table 1 and ln(NT-proBNP) were determined by linear regression analysis. We built a full model that included all variables considered in the bivariate analysis and performed a stepwise manual selection, retaining only variables with statistically significant individual associations (*p* < 0.05) in the multivariate model to determine which covariates were independently associated with ln(NT-proBNP).

Incidence rates for mortality with 95% confidence intervals were estimated using Poisson regression models and compared across categories of one-year change in hs-cTnT and NT-proBNP. To determine these categories with sufficient numbers of subjects in each stratum, the biomarker concentrations were dichotomized at each point in time (baseline and one-year follow-up). For hs-cTnT, the categories “undetectable” (hs-cTnT < 5.0 ng/L) and “detectable” (hs-cTnT ≥ 5.0 ng/L) were defined based on the LoD of the hs-cTnT assay. Based on the upper quartile of the baseline NT-proBNP concentrations, the categories “below” (NT-proBNP < 166.3 ng/L) and “above” (NT-proBNP ≥ 166.3 ng/L) of the upper quartile were defined. The change over time was then categorized into four groups based on each subject’s dichotomized baseline and follow-up categories.

The prognostic value of NT-proBNP at baseline (Model 2) and at one-year follow-up (Model 3) for mortality was assessed using Cox proportional hazard models after ln-transformation and addition to a base model (Model 1). The models were adjusted for well-established sociodemographic and clinical risk factors measured at baseline: age (years), sex (male, female), BMI (kg/m^2^), current smoking (yes, no), history of heart failure (yes, no), and diabetes mellitus (yes, no). In Model 4, the analysis was adapted for the covariates of Model 1 and simultaneously for continuous ln(NT-proBNP) concentrations at baseline and one-year follow-up. For the Cox models, time-dependent interactions of each variable with the natural logarithm of survival time were included to verify the assumption of proportional hazards. Because we found evidence of effect modification by sex in a previous study in a community-dwelling population of older adults [19], we also considered this in the current analysis. However, no significant interactions by sex were observed in the association between mortality and cardiac biomarkers (Model 4 interaction *p* values; ln(hs-cTnT): 0.497, baseline ln(NT-proBNP): 0.885, one-year ln(NT-proBNP): 0.964).

Given the distinctly skewed distribution of NT-proBNP, the temporal concentration changes were calculated as the percentage difference between the ln-concentrations at the follow-up and baseline, defined as

Relative Change = [(one-year ln(NT-proBNP) − baseline ln(NT-proBNP))/baseline ln(NT-proBNP)] * 100.(1)

We evaluated the relationship between these one-year relative changes in ln(NT-proBNP) as a continuous variable and mortality in Model 5, after adjustment for the one-year follow-up concentrations of ln(NT-proBNP) and the covariates of Model 1. These detailed analyses were performed only for NT-proBNP because the number of subjects with concentrations above the detection limit was too small for hs-cTnT, but hs-cTnT was considered as a covariate. Hazard ratio estimates were presented per standard deviation (SD) to increase comparability and readability of results.

To investigate model discrimination, Harrel-C statistics [20] were calculated with a 95% confidence interval for survival regression models with and without NT-proBNP and interval change. The increased differentiation value of changes in NT-proBNP concentration for mortality was assessed by the net reclassification improvement (NRI). This metric quantifies the difference in the probability that a subject belongs to predefined risk categories before and after the addition of a particular marker. The NRI for events and non-events by adding NT-proBNP measurements to the base model (Model 1) were calculated according to risk strata of <10%, 10 −< 20, and ≥20% of the estimated 10-year risk for mortality based on the methods of Pencina et al. [21].

In addition, dose-response relationships between baseline and follow-up NT-proBNP and survival outcome were plotted using restricted cubic splines with four nodes and after adjustment according to Model 4 using the variables described above. The reference for hazard ratios was the median concentration at each time point.

Statistical analysis was performed with SAS version 9.4 (SAS Institute, Cary, NC, USA) and R version 4.0.2 (R Foundation for Statistical Computing, Vienna, Austria).

## 3. Results

### 3.1. Subject Characteristics

Table 1 summarizes the baseline demographic characteristics of the 347 subjects included in the final analysis (median age 64.0 years, 61.7% female) with OA at the hip (57.1%) or knee (42.9%). The median value for BMI was 27.7 kg/m^2^ and 12.4% of the subjects reported current smoking at baseline. A history of diabetes mellitus, hypertension, myocardial infarction, and heart failure was documented for 8.9%, 47.6%, 3.2%, and 14.1% of the subjects, respectively.

Comparing the characteristics of the 347 subjects included with the 462 without adequate measurements, there were only few differences in demographics and most clinical measurements (Supplement Table S1). Subjects included were approximately 2 years younger (median 64.0 vs. 66.0 years) and had a higher eGFR (median 80.3 vs. 76.8 mL/min/1.73 m^2^). In addition, fewer subjects with a history of heart failure were in the current analysis population (14.1% vs. 22.5%) and a slightly higher proportion of subjects with a hip fracture was seen (57.1% vs. 48.1%).

### 3.2. Concentrations and Correlates of hs-cTnT and NT-proBNP

As further depicted in Table 1, the baseline concentrations of hs-cTnT were equal or higher than the detection limit (≥ 5.0 ng/L) in 108 subjects (31.1%) and the median concentration was lower than the detection limit at both measurement time points (< 5.0 ng/L). Almost all substrata of clinical characteristics showed a median hs-cTnT concentration below the detection limit at both baseline and follow-up. Only subjects with a history of myocardial infarction had a median concentration of 5.7 ng/L at baseline.

The median concentration of NT-proBNP was 95.0 ng/L at baseline and 101.6 ng/L at follow-up. The upper quartile of the NT-proBNP levels at baseline was 166.3 ng/L. This value was used as a reference value to categorize the change over time. In multivariate regression analysis, the variables that remained associated with higher NT-proBNP at both measurement time points were older age, female sex, a history of hypertension, and higher cystatin C concentrations (Supplement Table S2).

Baseline concentrations of NT-proBNP were correlated with baseline concentrations of hs-cTnT (age- and sex-adjusted Spearman’s partial correlation coefficient Rho = 0.14; *p* = 0.010) and with follow-up concentrations of NT-proBNP (Rho = 0.72; *p* < 0.001).

### 3.3. Categories of Changes in Biomarker Concentrations between Baseline and One-Year Follow-up and Mortality

During a median follow-up of 19.3 years (maximum 23.4 years) from the follow-up measurement, 209 (60.2%) of the subjects died. Overall, the rate was 3.5 per 100 person-years (95% CI 3.1–4.0). In deceased subjects, the median concentrations of NT-proBNP were higher at baseline (109.4 ng/L vs. 74.0 ng/L) and one-year follow-up (129.4 ng/L vs. 61.1 ng/L) when compared to other subjects. For hs-cTnT, all median values were below the detection limit (< 5.0 ng/L).

Incidence rates for mortality by categories of change from baseline are shown in Table 2. Subjects with initially low concentrations that increased above the reference value (hs-cTnT: 5.0 ng/L, NT-proBNP: 166.3 ng/L) from baseline to follow-up had a higher mortality rate per 100 person-years (hs-cTnT: 5.1 (95% CI 3.0–8.8), NT-proBNP: 4.4 (95% CI 3.1–6.3)) compared to subjects with biomarker levels that remained below the reference value (hs-cTnT: 2.7 (95% CI 2.2–3.3), NT-proBNP: 2.7 (95% CI 2.3–3.3)). The highest rate was found for subjects with concentrations above the reference value at both times (hs-cTnT: 6.2 (95% CI 4.6–8.3), NT-proBNP: 6.7 (95% CI 5.1–8.8)).

The categories of changes for hs-cTnT according to the threshold values 5.0 and 14.0 ng/L are shown in Supplement Table S3. The number of subjects with a hs-cTnT concentration of ≥ 14.0 ng/L was *n* = 13 at baseline and *n* = 17 at follow-up. Eight of these subjects were above 14.0 ng/L at both time points and had the highest mortality rate of all subgroups with 12.4 (95% CI 6.2–24.8) per 100 person-years.

### 3.4. Cox Proportional Hazard Models for Mortality

Table 3 shows the results of Cox regression analyses for mortality with NT-proBNP concentrations as ln-transformed continuous variables adjusted for conventional risk factors and the baseline ln(hs-cTnT) concentrations.

Both ln(NT-proBNP) values (baseline and follow-up) showed an increased hazard ratio (HR) for mortality when analyzed in separate models (Model 2 and Model 3). The addition of ln(NT-proBNP) measured at the one-year follow-up to the base model (Model 3) was associated with a slightly higher hazard for mortality compared to Model 2.

When including both baseline and one-year follow-up levels of ln(NT-proBNP) in Model 4, a one-SD increment of one-year follow-up ln(NT-proBNP) levels was associated with an HR of 1.31 (95% CI 1.04–1.66), while the baseline levels were no more associated with mortality (HR 1.08 (95% CI 0.86–1.37)).

Adding the relative one-year change in ln(NT-proBNP) to the model showed no association with mortality in the multivariate analysis (HR 0.94 (95% CI 0.78–1.14)), but the one-year follow-up value of ln(NT-proBNP) increased to an HR of 1.44 (95% CI 1.18–1.76) (Table 3, Model 5). Although the most recent one-year follow-up visit ln(NT-proBNP) level improved discrimination for deaths, information on NT-proBNP change did not add further prognostic value for mortality (C-statistic = 0.76 (95% CI 0.73–0.79)). The same applies to the calculated NRI parameters.

In addition, we repeated the Cox proportional hazards analysis with the same adjustments, truncating the follow-up period to 5, 10, and 20 years, and saw no essential changes in HRs with increasing follow-up period, documenting the robustness of the risk estimates based on NT-proBNP concentrations over the follow-up period (Supplement Table S4).

**Figure 2:**
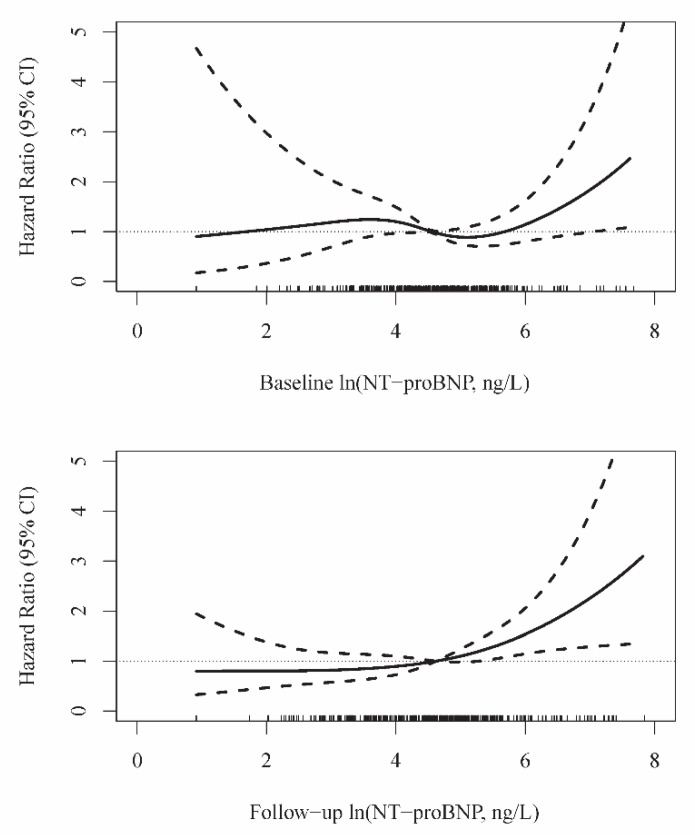
Relationship between NT-proBNP levels measured one year apart and mortality. Spline curves are adjusted for age, sex, BMI, current smoking, history of heart failure and diabetes mellitus, and baseline ln(hs-cTnT) (Table 3, Model 4).

Figure 2 shows restricted cubic spline curves that represent the dose-response relationship between ln(NT-proBNP) concentrations and mortality. The curves were consistent with findings from the Cox analyses, suggesting a significant risk increase for long-term mortality associated with higher one-year follow-up ln(NT-proBNP) concentrations, which were evaluated in the simultaneously adjusted model with baseline ln(NT-proBNP) levels (Table 3, Model 4).

## 4. Discussion

In this observational cohort study in subjects with OA who underwent arthroplasty, we found substantial changes in cardiac biomarkers measured at baseline and repeatedly one year later, especially for NT-proBNP, and an independent prognostic value for long-term mortality, particularly for the one-year measurement, while the baseline value showed no prognostic value when included. When considered alone, however, it showed a similar value compared to the one-year measurement. Therefore, no significant additional benefit of repeated NT-proBNP measurements was found in this cohort with normal NT-proBNP concentrations, facilitating the use of NT-proBNP as a stable prognostic marker.

OA is associated with cardiometabolic features (e.g., hypertension, diabetes mellitus, lipid metabolism disorders, and obesity). Studies suggest that subjects with OA have a higher prevalence of cardiovascular diseases (especially coronary artery disease and heart failure) than subjects without OA [12,13]. Furthermore, they subsequently also have an increased risk of premature mortality due to cardiovascular diseases [14,15]. Because of these shared cardiometabolic features, we used these well-established cardiovascular biomarkers in this OA population to investigate their prognostic value.

Several cohorts, predominantly with cardiovascular disease, have shown that higher hs-cTnT and NT-proBNP levels are associated with a higher risk of mortality [1,2,4,5,6,7,10,22]. A less conclusive picture emerges concerning the question of whether monitoring the temporal changes in concentrations of cardiac biomarkers in other cohorts could lead to a better prediction of mortality compared to a single measurement.

Both in subjects with initially low biomarker concentrations, whose concentration subsequently increased, and in subjects with values above the threshold value at baseline and follow-up, the categorical analysis of the change in biomarker concentration showed the highest mortality rates in the present study. The lowest mortality rate occurred in the category of subjects with initially low biomarker concentrations, whose concentration later also remained stable at low levels.

This is consistent with previous studies in which subjects were grouped into categories based on initial and follow-up biomarker concentrations relative to a threshold to analyze the change in concentration over time [23,24,25,26]. In contrast to the studies mentioned above, this group of subjects is not a primary cardiovascular population. Accordingly, the measured biomarker concentrations and thus the threshold value for classification in the high/low categories are low. Furthermore, the prevalence of hs-cTnT concentrations below the detection limit was also relatively high, resulting in an insufficient number of cases for an analysis of the hs-cTnT change as a continuous variable.

The increase in C-statistics achieved by adding NT-proBNP to other clinical risk predictors was very low. The addition of NT-proBNP measured at the one-year follow-up to a predictive model that included established risk factors and hs-cTnT resulted in better accuracy of risk classification than the addition of baseline concentrations. However, the effect when NT-proBNP was added was modest and resulted in an improvement of only the non-event, but not the event NRI. Concerning risk prediction based on the change in concentration, there was no effect on the long-term prediction of mortality risk when adjusted for the recent measurement of NT-proBNP.

Few previous studies have evaluated the prognostic utility of serial NT-proBNP measurements. Zile et al. found that a change in NT-proBNP is associated with a change in later cardiovascular mortality risk [26]. Masson et al. also suggested that serial determination of NT-proBNP concentration and categorical consideration of changes based on threshold values may be a superior strategy for risk stratification in patients with heart failure [24]. However, these results referred to populations with biomarker concentrations at higher levels. In this study of subjects with normal NT-proBNP levels, no additional value of repeated measurements could be shown. It therefore appears unnecessary to repeat NT-proBNP measurements in these patients after one year to obtain additional prognostic information. Baggen et al. came to a similar conclusion in a population of subjects with adult congenital heart disease (median age 33.0 years), who saw an additional prognostic value of serial NT-proBNP measurements most likely in subjects with elevated baseline concentrations [27]. They also discussed a simplified application and a possible cost reduction as possible positive effects of using NT-proBNP as a prognostic marker, which could result from longer intervals between measurements.

Physical activity is an important cornerstone in the relationship between OA and cardiovascular disease. On the one hand, it is an essential component in the prevention and treatment of cardiovascular disease. On the other hand, physical activity in subjects with OA is complicated by more severe pain on exercise, increased BMI, and lower functional level. However, the predictive value of biomarkers showed no significant change after replacement surgery within one year. Assuming an increase in physical activity with surgical joint replacement and yet a high prognostic value of cardiac biomarkers before surgery, effective treatment of cardiovascular risk factors continues to gain importance in the management of subjects with OA. However, our approach does not yet allow us to answer this interesting question, and the role of physical activity should be a topic of future research.

Strengths of the current study population include its detailed characterization at baseline to obtain information on established risk factors for death and cardiovascular disease. Further strengths are the long follow-up period of 20 years with a high degree of completeness and the relatively large number of deaths, which extends the statistical power for this outcome. Another strength is that biomarker concentrations could be measured in high quality with highly sensitive assays.

One of the limitations of the present study is the lack of detailed information about the long-term stability of the measured markers in frozen samples. However, in another study, we investigated the 8-year stability of NT-proBNP serum samples stored at −80 °C with a maximum of one thawing cycle and found an estimated recovery between 89.5% and 103% [28]. Additionally, the results are based on individual NT-proBNP measurements for both time points, so the daily variability could not be assessed. A further limitation is that only subjects with advanced hip or knee OA requiring unilateral joint replacement were included, which includes a selection of subjects with OA and affects the generalizability of the results. Moreover, the case numbers were not sufficient to perform survival analyses for hs-cTnT levels and their change between time points. Furthermore, the comorbidity (e.g., history of physician-diagnosed heart failure) was self-reported at baseline interview. Further details were not available and we were not able to consider the cause of death. Finally, it should be noted that only short-term (one-year) changes in cardiac biomarker concentrations were studied, although it may be more relevant to gain insight into changes over longer periods.

## 5. Conclusions

Detectable levels of NT-proBNP measured by a highly sensitive assay were present in the majority of older adults in this cohort, and higher levels reflect greater exposure to cardiovascular risk factors and heart disease. Regardless of these comorbidities, NT-proBNP concentrations were associated with all-cause mortality and might help to identify at-risk individuals early and take appropriate measures for effective treatment of cardiovascular risk factors in the management of patients with OA. However, no significant additional value of repeated NT-proBNP measurements could be detected in this cohort with measured NT-proBNP concentrations at baseline, which facilitates the use of a single measurement of NT-proBNP as a stable prognostic marker.

## Figures and Tables

**Table 1:** Baseline characteristics and median cardiac biomarker concentrations at baseline and one-year follow-up.

Characteristic	Category		Median		Median	
		Total	hs-cTnT, ng/L		NT-proBNP, ng/L	
		(*n* = 347)	Baseline	Follow-Up	Baseline	Follow-Up
Total		347 (100.0%)	<5.0	<5.0	95.0	101.6
Age, years		64.0 (12.0)				
Sex	Male	133 (38.3%)	<5.0	<5.0	68.2	61.9
	Female	214 (61.7%)	<5.0	<5.0	107.4	120.7
Localization of OA	Hip	198 (57.1%)	<5.0	<5.0	88.4	85.9
	Knee	149 (42.9%)	<5.0	<5.0	99.2	120.9
Body mass index, kg/m^2^		27.7 (5.6)				
Smoking status	Never	195 (56.2%)	<5.0	<5.0	101.7	118.7
	Former	109 (31.4%)	<5.0	<5.0	87.1	91.8
	Current	43 (12.4%)	<5.0	<5.0	69.8	74.2
Diabetes mellitus	No	316 (91.1%)	<5.0	<5.0	91.6	97.1
	Yes	31 (8.9%)	<5.0	<5.0	105.9	157.8
Hypertension	No	182 (52.4%)	<5.0	<5.0	76.9	70.4
	Yes	165 (47.6%)	<5.0	<5.0	113.2	139.8
Myocardial infarction	No	336 (96.8%)	<5.0	<5.0	95.0	99.8
	Yes	11 (3.2%)	5.7	<5.0	130.6	164.5
Heart Failure	No	298 (85.9%)	<5.0	<5.0	88.3	92.7
	Yes	49 (14.1%)	<5.0	<5.0	143.6	148.2
Cholesterol, mmol/L		5.7 (1.2)				
Triglyceride, mmol/L		1.5 (1.2)				
Uric Acid, mmol/L		313.0 (107.5)				
Cystatin C, mg/L		0.9 (0.2)				
eGFR, mL/min/1.73 m^2^		80.3 (26.9)				
hs-CRP, mg/L		2.3 (3.7)				
hs-cTnT, ng/L	<5.0	239 (68.9%)	<5.0	<5.0	86.1	89.7
	≥5.0	108 (31.1%)	7.5	<5.0	113.0	134.7
NT-proBNP, ng/L	<166.3	260 (74.9%)	<5.0	<5.0	72.0	72.5
	≥166.3	87 (25.1%)	<5.0	<5.0	252.4	257.1

Values are reported as *n* (percentage) or median (interquartile range).

**Table 2:** Categories of changes in biomarker concentrations between baseline and one-year follow-up and mortality (*n* = 347, 209 deaths).

Biomarker	Concentration, ng/L		Subjects	Deaths ^1^	Incidence Rate ^2^
	Baseline	Follow-Up	(*n* = 347)		(95% CI)
hs-cTnT	<5.0	<5.0	223 (64.3%)	109 (48.9%)	2.7 (2.2–3.3)
	≥5.0	<5.0	57 (16.4%)	43 (75.4%)	4.7 (3.5–6.3)
	<5.0	≥5.0	16 (4.6%)	13 (81.3%)	5.1 (3.0–8.8)
	≥5.0	≥5.0	51 (14.7%)	44 (86.3%)	6.2 (4.6–8.3)
NT-proBNP ^3^	<166.3	<166.3	220 (63.4%)	110 (50.0%)	2.7 (2.3–3.3)
	≥166.3	<166.3	24 (6.9%)	15 (62.5%)	3.7 (2.2–6.2)
	<166.3	≥166.3	40 (11.5%)	30 (75.0%)	4.4 (3.1–6.3)
	≥166.3	≥166.3	63 (18.2%)	54 (85.7%)	6.7 (5.1–8.8)

^1^ Percentages represent cumulative incidences in each category.

^2^ Cases per 100 person-years at risk of death.

^3^ The value of 166.3 corresponds to the upper quartile of the baseline NT-proBNP distribution.

**Table 3:** Cox proportional hazard models for mortality (*n* = 347, 209 Deaths).

Parameter/Statistic	Model 1	Model 2	Model 3	Model 4	Model 5
Hazard ratio for mortality, per SD (ln)					
NT-proBNP baseline		1.32 (1.13–1.55)		1.08 (0.86–1.37)	
NT-proBNP follow-up			1.39 (1.18–1.64)	1.31 (1.04–1.66)	1.44 (1.18–1.76)
Relative change					0.94 (0.78–1.14)
Model accuracy					
C-statistic	0.75 (0.71–0.78)	0.76 (0.72–0.79)	0.76 (0.73–0.79)	0.76 (0.73–0.79)	0.76 (0.73–0.79)
Event NRI	Reference	−0.02 (−0.13–0.12)	0.02 (−0.10–0.14)	0.00 (−0.13–0.13)	0.00 (−0.12–0.12)
Non-event NRI	Reference	0.03 (−0.03–0.08)	0.06 (−0.02–0.09)	0.06 (−0.02–0.09)	0.06 (−0.02–0.10)

Values are estimates with 95% confidence intervals.

Relative change is calculated as the percentage difference in ln(NT-proBNP) between baseline and one-year follow-up.

Note that subjects with hs-cTnT and NT-proBNP concentrations <5.0 ng/L had values imputed as 2.5 ng/L.

NRI, Net reclassification improvement (according to model-based 10-year risk categories of <10%, 10% –< 20%, or ≥20%).

Model 1—Age, sex, BMI, current smoking, history of heart failure and diabetes mellitus, and baseline ln(hs-cTnT).

Model 2—Model 1 + baseline ln(NT-proBNP).

Model 3—Model 1 + follow-up ln(NT-proBNP).

Model 4—Model 1 + baseline ln(NT-proBNP) + follow-up ln(NT-proBNP).

Model 5—Model 1 + follow-up ln(NT-proBNP) + relative change of ln(NT-proBNP).

## Data Availability

Due to ethical restrictions regarding data protection issues and the study-specific consent text and procedure, the data cannot be made publicly available, but data are available to all interested researchers upon request.

## Supplementary Materials

The following are available online at https://www.mdpi.com/2218-273X/11/2/230/s1, Table S1: Baseline characteristics of included and excluded subjects, Table S2: Correlates of ln-transformed NT-proBNP concentrations, Table S3: Categories of changes in hs-cTnT concentrations between baseline and one-year follow-up and mortality (*n* = 347, 209 Deaths), Table S4: Cox proportional hazard models for mortality with truncation of follow-up at 5, 10, and 20 years.
