# Supplementary material for: Repeated Measurements of Cardiac Troponin T and N-Terminal Pro-B-Type Natriuretic Peptide to Assess Long-Term Mortality Risk in Subjects with Osteoarthritis
Source: Biomolecules. 2021 Feb 5;11(2):230. doi: 10.3390/biom11020230 (PMC7915763; doi:10.3390/biom11020230)
Supplement: Supplementary file 1 [file biomolecules-11-00230-s001.pdf]

Supplementary Materials

Supplement Table S1. Baseline Characteristics of Included and Excluded Subjects

Characteristic	Category	Included (*n* = 347)	Excluded (*n* = 462)	Total (*n* = 809)	*p*-Value
Age, years		64.0 (12.0)	66.0 (11.0)	65.0 (12.0)	0.006
Sex	Male	133 (38.3%)	172 (37.2%)	305 (37.7%)	0.750
	Female	214 (61.7%)	290 (62.8%)	504 (62.3%)	
Localization of OA	Hip	198 (57.1%)	222 (48.1%)	420 (51.9%)	0.011
	Knee	149 (42.9%)	240 (51.9%)	389 (48.1%)	
Body Mass Index, kg/m^2		27.7 (5.6)	28.2 (5.2)	27.9 (5.4)	0.365
Smoking Status	Never	195 (56.2%)	274 (59.3%)	469 (58.0%)	0.556
	Former	109 (31.4%)	129 (27.9%)	238 (29.4%)	
	Current	43 (12.4%)	59 (12.8%)	102 (12.6%)	
Diabetes mellitus		31 (8.9%)	39 (8.4%)	70 (8.7%)	0.668
Hypertension		165 (47.6%)	250 (54.1%)	415 (51.3%)	0.065
Myocardial Infarction		11 (3.2%)	23 (5.0%)	34 (4.2%)	0.305
Heart Failure		49 (14.1%)	104 (22.5%)	153 (18.9%)	0.007
Cholesterol, mmol/L		5.7 (1.2)	5.7 (1.3)	5.7 (1.3)	0.855
Triglyceride, mmol/L		1.5 (1.2)	1.5 (1.1)	1.5 (1.1)	0.050
Uric Acid, mmol/L		313.0 (107.5)	315.4 (113.0)	315.4 (111.0)	0.375
Cystatin C, mg/L		0.9 (0.2)	0.9 (0.3)	0.9 (0.2)	0.142
eGFR, mL/min/1.73 m^2		80.3 (26.9)	76.8 (27.3)	78.2 (27.4)	0.034
hs-CRP, mg/L		2.3 (3.7)	2.6 (3.9)	2.5 (3.8)	0.427

Values are reported as n (%) or median (interquartile range). Differences between included and excluded subjects are analyzed using the Chi-square test for categorical data, One-Way ANOVA or Kruskal-Wallis for continuous data (depending on the distribution).

Supplement Table S2. Correlates of ln-transformed NT-proBNP Concentrations

Variable	Baseline ln(NT-proBNP)				Follow-up ln(NT-proBNP)			
	Bivariate		Multivariate		Bivariate		Multivariate	
	β-Coef.	*p*-Value	β-Coef.	*p*-Value	β-Coef.	*p*-Value	β-Coef.	*p*-Value
Age, years	**0.05**	**<0.001**	**0.04**	**<0.001**	**0.06**	**<0.001**	**0.04**	**<0.001**
Female Sex	**0.45**	**<0.001**	**0.30**	**0.005**	**0.53**	**<0.001**	**0.30**	**0.041**
Knee OA	0.09	0.426			**0.32**	**0.029**	−0.07	0.606
Body Mass Index, kg/m²	0.01	0.423			0.02	0.156		
Current Smoking	**−0.32**	**0.048**	0.10	0.522	−0.31	0.158		
Diabetes mellitus	0.17	0.363			0.49	0.055		
Hypertension	**0.51**	**<0.001**	**0.24**	**0.022**	**0.77**	**<0.001**	**0.49**	**<0.001**
Myocardial Infarction	0.47	0.126			0.81	0.050		
Heart Failure	**0.63**	**<0.001**	0.19	0.203	**0.58**	**0.005**	0.00	0.995
Cholesterol, mmol/L	0.03	0.512			−0.01	0.855		
Triglyceride, mmol/L	0.00	0.918			0.01	0.868		
Uric Acid, mmol/L	0.00	0.244			0.00	0.541		
Cystatin C, mg/L	**1.37**	**<0.001**	**0.79**	**0.003**	**1.53**	**<0.001**	**0.75**	**0.035**
eGFR, mL/min/1.73m²	**−0.01**	**<0.001**	0.00	0.355	**−0.02**	**<0.001**	0.00	0.756
ln(hs-CRP, mg/L)	0.09	0.068			0.12	0.078		

Multivariate linear regression analysis was used to identify the baseline variables (with bivariate $p < 0.05$) independently associated with NT-proBNP concentrations.

Supplement Table S3. Categories of Changes in hs-cTnT Concentrations between Baseline and one-year Follow-up and Mortality (*n* = 347, 209 Deaths)

hs-cTnT, ng/L		Subjects	Deaths*	Rate per 100 p-yr
Baseline	Follow-up	(*n* = 347)		(95% CI)
< 5.0	< 5.0	223 (64.3%)	109 (48.9%)	2.7 (2.2– 3.3)
< 5.0	5.0 –< 14.0	14 (4.0%)	11 (78.6%)	5.0 (2.8–9.1)
< 5.0	≥ 14.0	2 (0.6%)	2 (100.0%)	5.5 (1.4–22.0)
5.0 –< 14.0	< 5.0	53 (15.3%)	39 (73.6%)	4.5 (3.3–6.2)
5.0 –< 14.0	5.0 –< 14.0	35 (10.1%)	29 (82.9%)	5.5 (3.8–7.9)
5.0 –< 14.0	≥ 14.0	7 (2.0%)	6 (85.7%)	5.8 (2.6–12.9)
≥ 14.0	< 5.0	4 (1.1%)	4 (100.0%)	6.9 (2.6–18.4)
≥ 14.0	5.0 –< 14.0	1 (0.3%)	1 (100.0%)	5.6 (0.8–39.6)
≥ 14.0	≥ 14.0	8 (2.3%)	8 (100.0%)	12.4 (6.2–24.8)

* The percentages are the cumulative incidences in the respective category. p-yr indicates person-years at risk of death.

Supplement Table S4. Cox Proportional Hazard Models for Mortality with Truncation of Follow-up at 5, 10, and 20 Years

Parameter	Model 1	Model 2	Model 3	Model 4	Model 5
**Follow-up 5 years**					
Hazard ratio for mortality, per SD (ln)					
NT-proBNP Baseline		1.39 (0.83–2.32)		1.28 (0.57–2.87)	
NT-proBNP Follow-up			1.35 (0.78–2.36)	1.11 (0.48–2.58)	1.52 (0.74–3.11)
Relative Change					0.82 (0.36–1.87)
**Follow-up 10 years**					
Hazard ratio for mortality, per SD (ln)					
NT-proBNP Baseline		1.32 (1.02–1.72)		1.22 (0.80–1.87)	
NT-proBNP Follow-up			1.30 (0.99–1.73)	1.11 (0.72–1.72)	1.43 (0.99–2.05)
Relative Change					0.85 (0.56–1.30)
**Follow-up 20 years**					
Hazard ratio for mortality, per SD (ln)					
NT-proBNP Baseline		1.36 (1.15–1.61)		1.18 (0.92–1.51)	
NT-proBNP Follow-up			1.38 (1.16–1.64)	1.22 (0.95–1.58)	1.53 (1.22–1.92)
Relative Change					0.84 (0.66–1.07)

Values are estimates with 95% confidence intervals.
Relative change is calculated as the percentage difference in ln(NT-proBNP) between baseline and one-year follow-up.
Note that subjects with hs-cTnT and NT-proBNP concentrations <5.0 ng/L had values imputed as 2.5 ng/L.
Model 1—Age, sex, BMI, current smoking, history of heart failure and diabetes mellitus, and baseline ln(hs-cTnT).
Model 2—Model 1 + Baseline ln(NT-proBNP).
Model 3—Model 1 + Follow-up ln(NT-proBNP).
Model 4—Model 1 + Baseline ln(NT-proBNP) + Follow-up ln(NT-proBNP).
Model 5—Model 1 + Follow-up ln(NT-proBNP) + Relative Change of ln(NT-proBNP).
